# The magnetic-nanofluid heat pipe with superior thermal properties through magnetic enhancement

**DOI:** 10.1186/1556-276X-7-322

**Published:** 2012-06-20

**Authors:** Yuan-Ching Chiang, Jen-Jie Chieh, Chia-Che Ho

**Affiliations:** 1 Department of Mechanical Engineering, Chinese Culture University, No. 55, Hwa-Kang Rd., Yang-Ming-Shan, Taipei, 111, Taiwan; 2 Institute of Electro-Optical Science and Technology, National Taiwan Normal University, No.88, Sec.4, Ting-Chou Rd., Taipei, 116, Taiwan; 3 Department of Mechanical Engineering, National Central University, No.300, Jhongda Rd., Jhongli City, Taoyuan County, 32001, Taiwan

**Keywords:** magnetic nanofluids, thermal conductivity, slug, vapor

## Abstract

This study developed a magnetic-nanofluid (MNF) heat pipe (MNFHP) with magnetically enhanced thermal properties. Its main characteristic was additional porous iron nozzle in the evaporator and the condenser to form a unique flowing pattern of MNF slug and vapor, and to magnetically shield the magnet attraction on MNF flowing. The results showed that an optimal thermal conductivity exists in the applied field of 200 Oe. Furthermore, the minor thermal performance of MNF at the condenser limited the thermal conductivity of the entire MNFHP, which was 1.6 times greater than that filled with water for the input power of 60 W. The feasibilities of an MNFHP with the magnetically enhanced heat transfer and the ability of vertical operation were proved for both a promising heat-dissipation device and the energy architecture integrated with an additional energy system.

## Background

With the gradually increasing development of dense electrical circuit electronics, several aspects were considered for the typical improvements on a heat pipe, referred to as a heat superconductor. Nanofluids with excellent thermal conductivity [1] were applied as the working fluids (WFs) of traditional heat pipes to enhance the thermal performance [2,3]. However, either high-cost metal nanoparticles solved in water were used as nanofluids or these heat pipes were operated only in a horizontal or limited tilt arrangement, rather than the vertical arrangement, in which their thermal performance deteriorates because nanoparticles always accumulate on the evaporator of heat pipes with evaporation converting water to vapor.

An oscillating heat pipe [4-6] with an additional heat transfer mechanism of conventional force through the interval liquid slug and vapor bubble beyond that of the phase change can overcome the deposition problem. Its closed loop suppresses the entrainment limit, that is, the shear force between condensing and evaporating flow in high-input power [7]. However, its multi-turn architecture limits its integration with electronics.

Among low-cost metal-oxide nanofluids [8-10], magnetic nanofluids (MNFs) have the magnetically enhanced thermal conductivity [8,9] to compete with metal nanofluids. However, the common disadvantage of developed MNFHPs in overcoming the deposition problems is the extra-induced problem of magnetic manipulation resulting from unsuitable designs of either active or complex magnetic fields [11,12]. To avoid the extra power consumption from actively controlling the MNF flowing, a novel MNFHP in a closed-loop architecture was proposed for several main objects: the first was the flowing pattern of the interval vapor moving the MNF slug; the second was the superior suppression of the entrainment limit; and the third was the expansible ability for the integration with some energy storage [13].

## Methods

**Figure 1:**
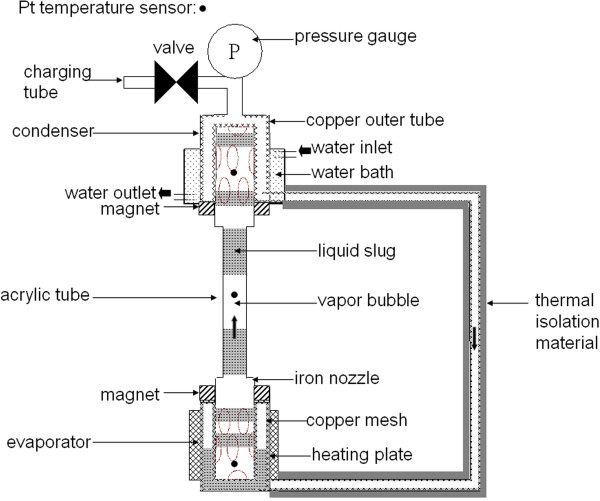
The closed-loop type of a MNFHP.

Figure 1 shows a closed-loop type of MNFHP. Its main difference from traditional heat pipes is the inner structure, that is, the additional iron porous nozzle in both evaporator and condenser, and magnets on its evaporator and condenser. The iron porous nozzle suppresses magnetic attraction for MNF flowing and forms the flowing pattern of the interval flowing of MNF slug and vapor bubble. Similarly, copper meshes sintered on whole inner surface were also used to transport MNFs by the capillary force against the magnetic attraction.

**Figure 2:**
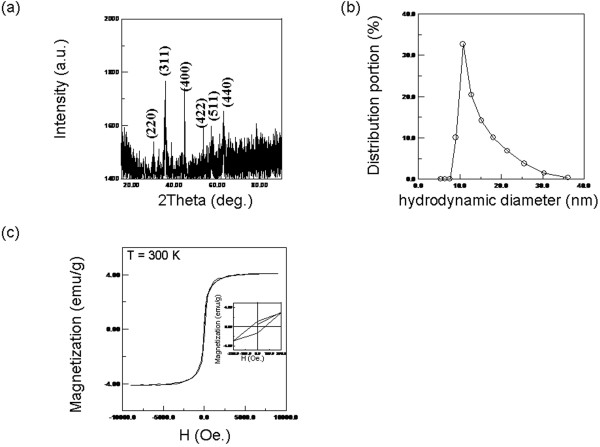
**Characterization of MNFs.** (**a**) The crystallines of magnetic nanoparticles, (**b**) hydrodynamic diameters of the magnetic nanoparticles, and (**c**) the magnetization properties.

The used WFs included water as the reference and different concentrations of MNFs composed of a water solvent and magnetic nanoparticles of Fe_3_O_4_ (Taiwan Advanced Nanotech Corp., Taoyuan,Taiwan). The crystallines of magnetic particles were analyzed, as shown in Figure 2a, using a powder X-ray diffractometer (D-500, Siemens Corp., Belgium, Germany) with Cu-Kα radiation at a wavelength of 0.15418 nm. Figure 2a shows that the phases of Fe_3_O_4_ agreed with the standard diffraction spectrum (JCPDS no. 65–3107).

The size distribution of the magnetic particles was investigated, as shown in Figure 2b, using dynamic laser scattering (Nanotrac 150, Microtrac Corp., PA, USA). The average hydrodynamic diameter was 10 nm ± 14 nm. The diameter that was detected using dynamic laser scattering is a hydrodynamic diameter because detecting the Brownian motion of the particles was conducted by probing the Doppler frequency shift of the scattered light with respect to the incident light.

The magnetization properties of MNFs were examined using a vibration sample magnetometer (Model 4500, EG&G Corp, California, USA). The concentration of MNFs was diluted with deionized (DI) water. For example, the magnetization of 4 emu/g, equal to 0.8% in volumetric fraction, was obtained (Figure 2c).

### Measurements and analysis models

In designing the decrease of the total thermal resistance (*R*_WF_) of an MNFHP, the major thermal resistance of the evaporator or condenser was reduced using MNFs with excellent thermal conductivity. The thermal resistance related with the MNF flowing between an evaporator and a condenser was accomplished by specifically configuring an MNFHP. Therefore, the improvement of the heat transfer in an evaporator or a condenser with magnetic fields was initially evaluated by testing the evaporator or condenser.

**Figure 3:**
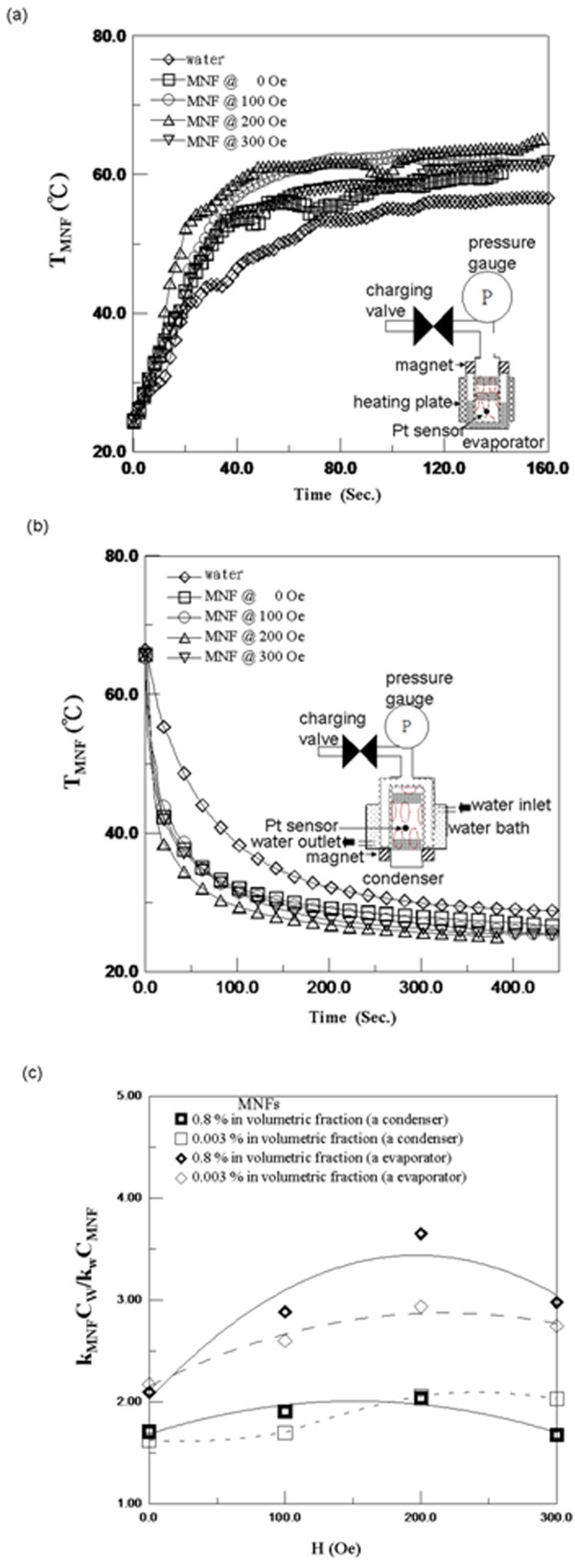
**Characterization of the subunit.** (**a**) The evaporator in the heating *Q*_sensible_ procedure, (**b**) the condenser in the cooling *Q*_sensible_ procedure, and (**c**) with the *H*-dependent *k*_MNF_C_W_ / *k*_W_*C*_MNF_.

Both the evaporator and condenser were composed of a copper tube and a porous iron nozzle, as shown in the inset of Figure 3a,b. By connecting the stuffing parts, such as the charging valve and pressure gauge, the evaporator or the condenser was filled with the same amount of WFs. By setting the temperature of the heating or cooling sources (*T*_s_), the temperature variation of WFs, *T*_WF_, was measured within different magnetic fields, for example, Figure 3a,b for the MNF of 0.8% in volumetric fraction. In addition, the heat transfer (*Q*) contributed to WFs in two types: latent heat (*Q*_latent_) and sensible heat (*Q*_sensible_). *Q*_latent_ was the same regardless of whether the WFs were MNFs or water, but *Q*_sensible_ was the key property for distinguishing MNFs from water in thermal performance. Therefore, *Q*_sensible_ is representatively discussed using a one-dimensional model ($Q_{\text{sensible}}=k_{\text{WF}}A_{\text{WF}}\frac{\left(T_{\text{tube}}-T_{\text{WF}}\right)}{d_{\text{WF}}}=\rho_{\text{WF}}V_{\text{WF}}C_{\text{WF}}\frac{dT_{\text{WF}}}{dt}$). Here, *Q*_sensible_ is related to the thermal conductivity (*k*_WF_), temperature (*T*_WF_), density (*ρ*_WF_), specific heat (*C*_WF_), thickness (*d*_WF_), volume (*V*_WF_), cross-area (*A*_WF_) of the WFs between the outer surface of the iron nozzle and the inner surface of the evaporator or the condenser, and the tube temperature (*T*_tube_). *T*_tube_ is the same as *T*_s_ with the reasonable assumption of low thermal resistance, and *t* is the time after applying *Q*.

This one-dimensional model can be solved as *T*_WF_ (TWF=(TWF.0−Ttube)e−(kWFAWFdWFρWFVWFCWF)t+Ttube) from the initial temperature of the WFs (*T*_WF,0_). Here, the time constant (*τ*) of *T*_WF_ is the reciprocal of kWFAWFdWρWFVFFCWF. Furthermore, *τ* is obtained according to the fitting of the *T*_WF_ variation with time, as shown in Figure 3a,b. To examine the influences of WFs and applied magnetic field (*H*) on the conductivity of an evaporator or a condenser, the thermal indicator of *k*_WF_ / *C*_WF_ was analyzed based on *T*_WF_ under the reasonable assumption of the similar densities for water and MNFs for the test concentration of 0.8% and 0.003% in volumetric fraction. Hence, the enhanced ratio of *k*_WF_ / *C*_WF_ was defined as *k*_MNF_*C*_W_ / *k*_W_*C*_MNF_ to examine the effect of WFs by comparing *C*_WF_ and *k*_w_ of DI water.

Consequently, to determine the total thermal performance of the entire MNFHP, the proposed scheme was modified with some additional parts to measure the pressure and temperature, as well as the WF stuffing (Figure 1). For different WFs or applied fields, the operation conditions were the same. In addition to the observation of the flowing pattern through the acrylic tube between the evaporator and condenser, the *R*_WF_ of the MNFHP, defined as $\left(T_{\text{evap}}-T_{\text{cond}}\right)/P$, was analyzed. Here, *T*_evap_ and *T*_cond_ are the temperatures of WF in the evaporator and condenser, and the heat transfer of the heat pipes, *P*, is obtained via the thermal exchange of the cooling water surrounding the condenser. Similarly, the enhanced thermal resistance ratio (*R*_MNF_ / *R*_W_) was used to compare the effect of WFs, where *R*_MNF_ and *R*_W_ were under the MNFHP filled with MNFs and water, respectively.

## Results and discussion

Regarding the analysis of the thermal properties of an evaporator or a condenser, Figure 3a,b shows that *T*_WF_ increased or decreased exponentially over time for WFs of water or MNFs at different fields. For MNFs, both $\left|T_{\text{WF},f}-T_{\text{tube}}\right|$ and *τ* decreased with *H*s to optimal values at *H* of 200 Oe, where *T*_WF,f_ was the final temperature of WF. Furthermore, both $\left|T_{\text{WF},f}-T_{\text{tube}}\right|$ and *τ* of MNFs were smaller than those of water. Based on the one-dimensional model, $\left|T_{\text{WF},f}-T_{\text{tube}}\right|$ decreased with the increase of *k*_WF_ under the same final heat transfer, *Q*_sensible,f_, because *Q*_sensible,f_ is equal to the system of heat loss. In addition, *τ* could be evaluated according to the dominator of *C*_WF_ / *k*_WF_. Therefore, the variations of $\left|T_{\text{WF},f}-T_{\text{tube}}\right|$ and *τ* were related to the thermal parameters of *k*_WF_ and *C*_WF_.

Furthermore, considering the variation of *H*s and MNF concentrations in both the evaporator and condenser, Figure 3c indicates that all *k*_MNF_*C*_W_ / *k*_W_*C*_MNF_ increased with *H*s and have an optimal value at a specific *H*. The optimal phenomenon was not significant for low concentrations of MNFs. Moreover, the *k*_MNF_*C*_W_ / *k*_W_*C*_MNF_ of the evaporator was larger than that of the condenser. The reasons were explained in *k*_WF_ and *C*_WF_ at different *H*s and temperatures.

For magnetic nanoparticles of Fe_3_O_4_*k*_MNF_ was larger than *k*_W_ in proportion to the concentration [8,9]. *k*_MNF_ increased with *H*s and had an optimal value at a specific *H*[8,9]. The specific field increased, whereas the concentration was lower [8]. Because of the formation of magnetic clusters, *C*_MNF_ decreased with *H*s, becoming smaller than 300 Oe [14]. Because the origin of mechanism is the same as that of *k*_MNF_, there was possibly an optimal value for the variation of *C*_MNF_ with *H*s. This was valid for other *H*-dependent optical properties of MNFs based on this mechanism [15]. Regardless, *k*_MNF_ / *C*_MNF_ had an optimal value at a specific *H*, but *k*_W_ / *C*_W_ maintained the same value for all *H*s.

In WF temperature, *k*_MNF_ decreased as the temperature rose [16], but *k*_W_ almost remained the same [17]. Similarly, *C*_MNF_ decreased slightly as the temperature rose, but *C*_W_ almost remained the same [14]. Effectively, *k*_MNF_*C*_W_ / *k*_W_*C*_MNF_ is smaller at high temperatures than at low temperatures if the effect of *C*_MNF_ is smaller than that of *k*_MNF_.

Therefore, for the enhanced time of heat transfer in an evaporator or a condenser, *k*_MNF_*C*_W_ / *k*_W_*C*_MNF_, as depicted in Figure 3c, achieved up to 3.5 and 1.6, separately, under the optimal condition of MNFs of 0.8% in volumetric fraction at 200 Oe.

**Figure 4:**
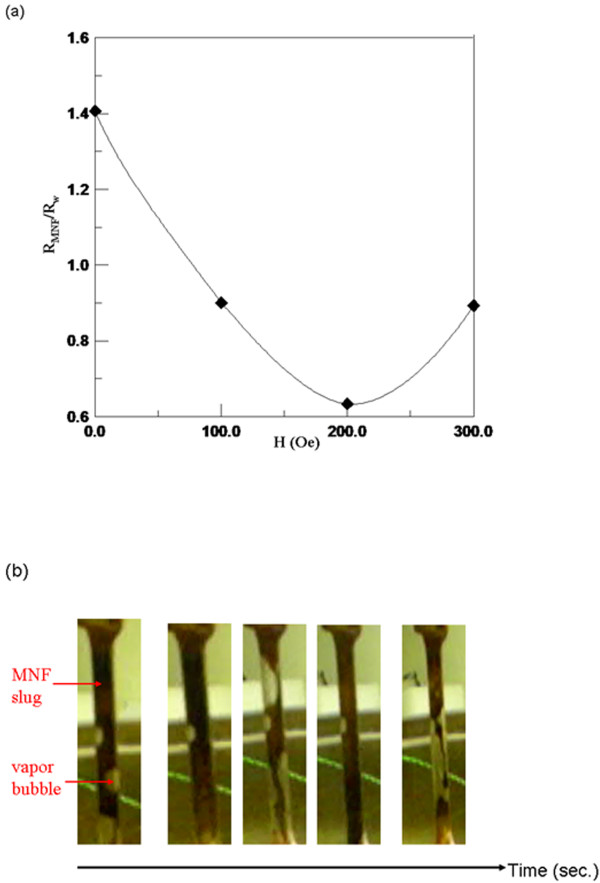
**Characterization of a MNFHP filled with MNFs of 0.8% in volumetric fraction.** (**a**) The *H*-dependent *R*_MNF_ / R_W_, and (**b**) the flowing pattern at 200 Oe.

Furthermore, by filling MNFs with 0.8% in volumetric fraction into an entire MNFHP, thermal performance of *R*_MNF_ / *R*_W_ at different *H*s was analyzed in Figure 4a, indicating that *R*_MNF_ / *R*_W_ was less than 1 at all *H*s except zero field. The worst *R*_MNF_ at zero fields was unexpected according to *k*_MNF_*C*_W_ / *k*_W_*C*_MNF_ of a single evaporator or a condenser in the *Q*_sensible_ test, larger than 1. Possibly, MNF with the high concentration easily resulted in the deposition of magnetic nanoparticles on the tube wall of the evaporator. Conversely, for the smaller *R*_MNF_ / *R*_W_ at the nonzero field, the deposition phenomenon was possibly avoided. The reason was that the more violent scour on the tube walls of a condenser and an evaporator caused by the faster MNF flowing under higher *k*_MNF_*C*_W_ / k_W_C_MNF_ might decrease the MNP deposition. The minimum of *R*_MNF_ / R_W_ occurred at the same as 200 Oe with *k*_MNF_*C*_W_ / *k*_W_*C*_MNF_ of a single evaporator and condenser in Figure 3c. Furthermore, *R*_MNF_ / *R*_W_ of the entire MNFHP could be viewed as the ratio of the effective thermal conductivity (*k*_W,MNFHP_ / *k*_MNF,MNFHP_) with a simple conduction model in that the thermal resistance *R* was equal to *k*_WF,MNFHP_*A*_tube_ / *d*_tube_, where the effective thermal conductivity of the entire MNFHP filled with some WF (*k*_WF,MNFHP_), the cross area (*A*_tube_) and diameter (*d*_tube_) of the tube are in form, derived from the one-dimensional conduction model. Therefore, the minimal value of *R*_MNF_ / *R*_W_ of approximately 0.6 was equal to this maximal value of *k*_MNF,MNFHP_ / *k*_W,MNFHP_ of 1.6. This value is similar with the smaller optimal value of *k*_MNF_*C*_W_ / *k*_W_*C*_MNF_ in a single condenser rather than that of the larger single evaporator. It could be concluded that the thermal performance of an entire MNFHP was limited by the smaller one because of the series assembly of an evaporator and a condenser, as well as a minor thermal resistance of the flowing mechanism.

Moreover, under the optimal condition of MNFs of 0.8% in volumetric fraction at 200 Oe, the total thermal performance of the entire MNFHP under the optimal condition achieved the maximal *P* of 60 W and the flowing pattern of the MNF slug and vapor bubble, as shown in the photos at the time interval of 1 sec in Figure 4b, was observed in the acrylic tube between the evaporator and the condenser, thus confirming that the flowing pattern was the design.

## Conclusion

This study developed an MNFHP in a closed-loop scheme with magnetically enhanced thermal properties, the flowing pattern of the interval liquid slug and vapor bubble, and the separated paths of evaporated fluids and condensed fluids. The feasibility of an MNFHP with magnetically enhanced thermal performance is valid. The enhanced thermal conductivity ratio of an MNFHP showed that the entire MNFHP was limited by the *k*_MNF_*C*_W_ / *k*_W_*C*_MNF_ of a condenser subunit. This was useful in designing an MNFHP.

## Abbreviations

DI, deionized; MNFs, magnetic nanofluids; MNFHP, magnetic-nanofluid heat pipe; WFs, working fluids.

## Competing interests

The authors declare that they have no competing interests.

## Authors’ contributions

YCC conducted the experiment. JJC provided the idea and proofread the manuscript. CCH provided technical support for the manufacturing equipment. All authors read and approved the final manuscript.

## Authors’ information

YCC has been an assistant professor in the Department of Mechanical Engineering, Chinese Culture University. His research interests include thermodynamics, heat transfer in electronics, and energy engineering. JJC has been an associate professor in the Institute of Electro-Optical Science and Technology, National Taiwan Normal University. His research interests include photonics, energy, and biomagnetism. CCH was a PhD degree candidate in the Department of Mechanical Engineering, National Central University. He had rich practical experiences of related manufacturing equipment in addition to his major in micro- and nano-scale manufacturing.
